# Early parental deprivation during primate infancy has a lifelong impact on gene expression in the male marmoset brain

**DOI:** 10.1038/s41598-023-51025-z

**Published:** 2024-01-03

**Authors:** Haruka Shinohara, Makiko Meguro-Horike, Takashi Inoue, Miyuki Shimazu, Machiko Hattori, Hitoshi Hibino, Kazumasa Fukasawa, Erika Sasaki, Shin-ichi Horike

**Affiliations:** 1 Division of Integrated Omics Research, Research Center for Experimental Modeling of Human Disease, Kanazawa University, Kanazawa, 920-0934 Japan; 2 Department of Marmoset Biology and Medicine, Central Institute for Experimental Animals, Kawasaki, 210-0821 Japan; 3 United Graduate School of Child Development, Osaka University, Kanazawa University, Hamamatsu University School of Medicine, Chiba University and University of Fukui, Kanazawa, 920-8640 Japan; 4 Yaotsu Breeding Center, CLEA Japan, Inc, Yaotsu-cho, Kamo-gun, Gifu, 505-0307 Japan

**Keywords:** Gene expression, Neurological disorders, Neurodevelopmental disorders, ADHD, Autism spectrum disorders, Transcriptomics

## Abstract

Adverse early life experiences are well-established risk factors for neurological disorders later in life. However, the molecular mechanisms underlying the impact of adverse experiences on neurophysiological systems throughout life remain incompletely understood. Previous studies suggest that social attachment to parents in early development are indispensable for infants to grow into healthy adults. In situations where multiple offspring are born in a single birth in common marmosets, human hand-rearing is employed to ensure the survival of the offspring in captivity. However, hand-reared marmosets often exhibit behavioral abnormalities, including abnormal vocalizations, excessive attachment to the caretaker, and aggressive behavior. In this study, comprehensive transcriptome analyses were conducted on hippocampus tissues, a neuroanatomical region sensitive to social attachment, obtained from human hand-reared (N = 6) and parent-reared male marmosets (N = 5) at distinct developmental stages. Our analyses revealed consistent alterations in a subset of genes, including those related to neurodevelopmental diseases, across different developmental stages, indicating their continuous susceptibility to the effects of early parental deprivation. These findings highlight the dynamic nature of gene expression in response to early life experiences and suggest that the impact of early parental deprivation on gene expression may vary across different stages of development.

## Introduction

Early life experiences, such as parent loss, neglect, and child abuse, are well-established risk factors for neurological disorders later in life, including depression, anxiety disorders, and post-traumatic stress disorder^1,2^. Various experiences contribute to neural plasticity and neurophysiological processes involved in learning and memory throughout life. Conversely, many types of adverse experiences have long-term negative consequences on cognitive, emotional, and behavioral development^3,4^. Adequate parental care during childhood is indispensable for developing neuronal networks with neural plasticity^5^. Previously, a study demonstrated the influence of contact comfort on primate development^6^. In this study, infant rhesus monkeys were separated from their mothers and some infants were housed in isolated cages away from peers. These monkeys exhibited disturbed behavior, such as blank stares, circling within their cages, and self-mutilation due to social isolation. Upon reintroduction to the group, the isolated infants displayed uncertainty in their interactions—many remained isolated from the group, and some even succumbed after refusing to eat. Thus, this early period, characterized by heightened sensitivity to the environment, plays a crucial role in establishing lifelong patterns of physiological reactivity and behavior. Specifically, the early developmental period [postnatal day 1 to day 28 in the common marmoset (*Callithrix jacchus*)], characterized by intensive mother-infant contact, is essential for adapting to early home environments through maternal care, which involves skin-to-skin contact between a mother and her newborn^7^. However, the molecular mechanisms underlying the impact of adverse childhood experiences on neurophysiological systems throughout life remain incompletely understood^8^. Several rodent models have been instrumental in investigating these mechanisms and have revealed that adverse experiences, resulting in heightened responsiveness of the hypothalamic–pituitary–adrenal (HPA) axis, can induce behavioral anxiety through epigenetic programming of glucocorticoid receptor (*GR*) expression^9–11^. These findings highlight the significant impact of adverse experiences, such as mother-infant separation, in instigating epigenetic alterations in infants, leading to enduring effects on gene expression due to maternal care. However, rodents differ notably from humans in aspects of parental care, secretion systems, stress responses, brain structures, and neurodevelopment, which emphasizes the critical role of primate models in corroborating the implications derived from rodent models^12,13^. For instance, a study demonstrated the profound influence of familial interactions on primate development by examining the consequences of parental deprivation in marmosets, employing the postnatal environmental manipulation paradigm initially established in rats^14^. In common marmosets, early life experiences resulting from repeated social isolation during infancy triggers acute stress responses, activating the HPA axis and potentially inducing neurodevelopmental changes. Notably, the repeated daily isolation of marmoset infants significantly elevates cortisol, epinephrine, and norepinephrine levels compared to controls^15,16^. Furthermore, consistent findings indicating reduced hippocampal expression of the mineralocorticoid receptor (*MR*) and the glucocorticoid receptor (*GR*) suggest lasting effects of this stress^17^. Therefore, primate studies provide crucial insights that complement rodent research, despite differences in the postnatal state of the HPA system between these species. These investigations propose that early life experiences prompting acute stress responses disrupt physiological brain balance through the HPA system. Conversely, the classic Harlow studies presented pivotal evidence, highlighting the fundamental importance of parent–child attachment relationships and the essential role of maternal touch in infant development. His experiments underscored the significance of fulfilling emotional needs in infants for healthy development, suggesting that love, affection, and comfort are indispensable for infants to grow into healthy adults^18^.

Given the existing evidence on the influence of early-life experiences, such as mother-infant separation, on infant physiology, cognition, and social behaviors across various species^9,10,19–27^, we chose the common marmoset, a New World monkey, as our model for investigating the impact of early parental deprivation. The common marmoset is a genetically manipulatable primate that has been used as a suitable model to understand the molecular basis of neurophysiological processes. Their developmental rate and reproductive capacity make them powerful models for genetic engineering^28^. Moreover, the common marmoset provides valuable insights into the study of parental infant care, which shares similarities with human caregiving. Marmosets live in family units and have excellent vocal communication, with mothers, fathers, and other kin participating in infant-rearing and sharing food with the offspring^29^. In situations where multiple offspring are born in a single birth, there is indeed competition for limited resources like milk from the mother. Consequently, the weaker or smaller offspring may not receive enough nourishment, making them more vulnerable and prone to failing to thrive, ultimately leading to their death. To address this issue, caretakers in animal facilities often opt to human hand-rear the weaker and smaller neonates when multiple offspring are born in a single birth. This intervention helps to ensure that the limited resources and care available can be directed towards a smaller number of offspring, increasing their chances of survival. However, it has been noted that human hand-reared marmoset neonates exhibit behavioral abnormalities that are not observed in parent-reared neonates^19,20^. On a related note, studies have shown that peer-reared rhesus macaques which develop in the absence of their parents exhibit behavioral abnormalities, such as insecure attachment and heightened levels of anxiety^21^. These observations highlight the critical role of social relationships and attachment to parents in early development, serving as essential factors for infants to mature into healthy adults as described previously^7^. Notably, decreased expression of the *OXTR* gene and a reduced abundance of H3K4me3 modification in the promoter region of the *OXTR* gene were observed in the hippocampus of macaques exposed to disrupted maternal care^21^. These findings suggest the involvement of the oxytocin system in response to mother-infant separation.

**Figure 1:**
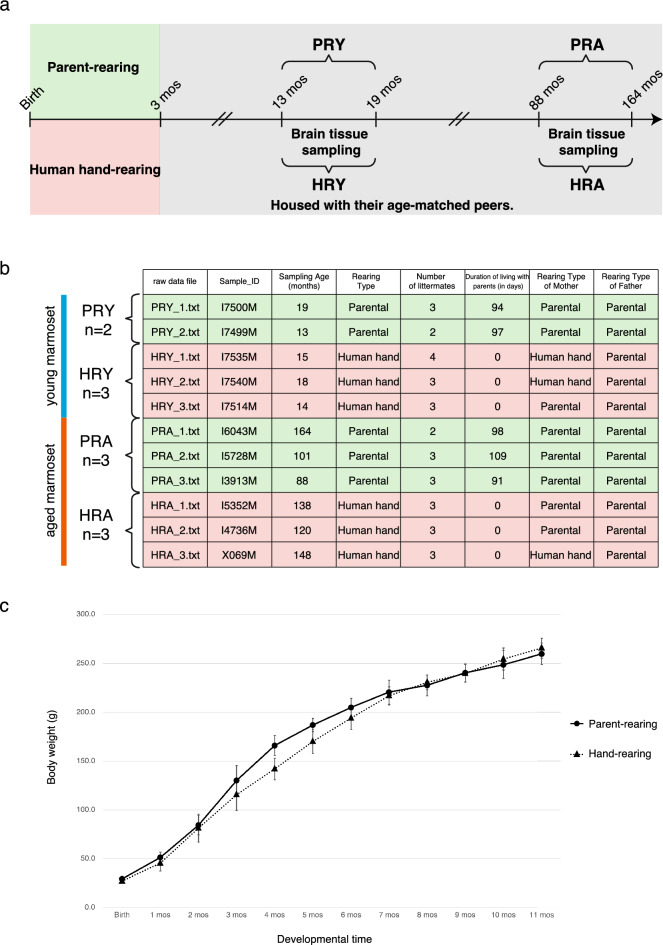
Overview of the experimental workflow & Monthly body weight measurements. **(a)** Overview of the experimental workflow. *HRY* *h*and-*r*eared *y*oung marmoset, *PRY* *p*arent-*r*eared *y*oung marmoset, *HRA* *h*and-*r*eared *a*ged marmoset, *PRA* *p*arent-*r*eared *a*ged marmoset. **(b)** Sample information. **(c)** Monthly body weight measurements of parent-reared marmosets and human hand-reared marmosets were taken for eleven months, starting from the day of their birth.

Thus, it is evident that early life experiences, such as human hand-rearing or mother-infant separation, have the potential to impact the neurophysiological systems and gene expression patterns in infant brains. Therefore, we aimed to investigate the hypothesis that human hand-rearing induces alterations in gene expression within the brains of infants, and that these changes persist into adulthood with potential epigenetic alterations. Previous research has indicated a decrease in gene expression of the *5-HT1A* receptor gene in the hippocampus of marmoset infants subjected to early parental deprivation, as determined by in situ hybridization^22^. However, whether human hand-rearing specifically influences global gene expression patterns in the brains of marmoset infants has yet to be fully elucidated. In this study, comprehensive transcriptome analyses were conducted on hippocampus tissues, a neuroanatomical region sensitive to social attachment, obtained from human hand-reared (N = 6) and parent-reared male marmosets (N = 5) at distinct developmental stages (Young: 13 to 19 months and Aged: 88 to 164 months) (Fig. 1a). Thus, investigating the effect of early parental deprivation on gene expression and potential epigenetic changes could be crucial in understanding the role of early mother–child attachment at a genetic level.

## Results

### Early parental deprivation model

The primary objective of this study was to explore the short-term and long-term consequences of early parental deprivation by analyzing gene expression in the hippocampus tissues of eleven male marmosets. Among the marmosets included in the study, six were subjected to human hand-rearing, while the remaining five were reared by their parents. After birth, the marmosets underwent either human hand- or parental rearing for a duration of 3 months (Fig. 1a). Subsequently, they were housed together with their age-matched peer. To categorize the marmosets based on age, those falling within the age range of 13 to 19 months were classified as Young Marmosets, while those within the age range of 88 to 164 months were designated as Aged Marmosets (Fig. 1b). The gene expression alterations observed in the Young Marmosets due to human hand-rearing in the initial 3 months post-birth were classified as short-term consequences. Conversely, the gene expression alterations observed in Aged Marmosets were characterized as long-term consequences. To account for the potential impact of different feeding patterns and nutritional compositions on brain development, monthly weight measurements were conducted on both human hand-reared and parent-reared marmosets starting from birth (Fig. 1c). The results showed slight differences in body weight at 4 months of age between the two groups. Marmosets that underwent human hand-rearing during the first 3 months after birth showed distinct variations in body weight compared to those that received parental rearing. However, after reaching 7 months of age, no significant differences in body weight were observed among the marmosets, suggesting that their nutritional needs were adequately met for normal brain development.

In this study, while we did not conduct specific quantitative behavioral analysis or vocal measurements, notable behaviors were observed. They exhibited increased aggression or demanding behavior towards caretakers, suggesting potential social and behavioral implications of early parental deprivation, as previously described^19,20^. Furthermore, their vocal development seemed to resemble that of an immature infant. These observations underscore the influence of early-life experiences on both social conduct and vocalization in human hand-reared marmosets, as elaborated earlier^20^.

Taken together, we suggested that while there may be initial differences in body weight due to variations in feeding patterns, the nutritional needs of human hand-reared marmosets can be sufficiently met for normal brain development. However, the observed behavioral and vocal immaturity in human hand-reared marmosets indicates potential long-term effects of early parental deprivation on social behavior and communication skills.

### The pathway of neuroactive ligand-receptor interaction may play a significant role in the short-term consequences of early parental deprivation

**Figure 2:**
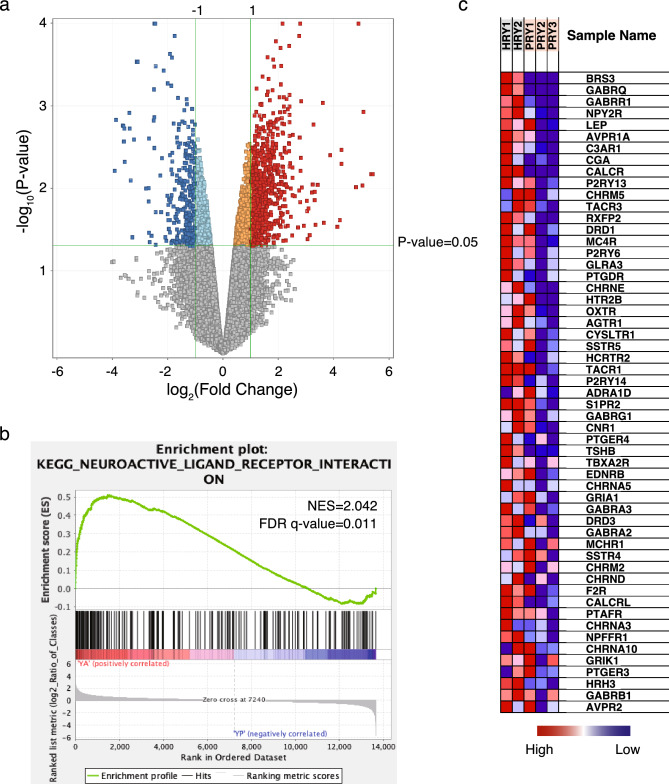
The pathway of neuroactive ligand-receptor interaction may play a significant role in the short-term consequences of early parental deprivation. **(a)** A volcano plot was generated to visualize the results of the microarray analysis comparing human hand-reared young marmosets to parent-reared young marmosets. In the plot, 425 genes that exhibited upregulation in the human hand-reared marmosets were shown in red color (fold change > 2.0, P < 0.05). On the other hand, 188 genes that displayed downregulation were shown in blue color (fold change < −2.0, P < 0.05). **(b)** Gene Set Enrichment Analysis (GSEA) of the neuroactive-ligand receptor interaction pathway enriched in human hand-reared marmosets compared to parent-reared young marmosets. **(c)** Heat map was visualized neuroactive ligand-receptor interaction pathway genes. *HRY* *h*and-*r*eared *y*oung marmoset, *PRY* *p*arent-*r*eared *y*oung marmoset, *red* higher expression, *blue* lower expression.

To investigate the molecular mechanisms underlying the short-term consequences of early parental deprivation in human hand-reared marmosets, a detailed analysis of gene expression changes in hippocampus tissues was conducted. It is hypothesized that early parental deprivation can influence the neuronal gene expression program by changing persistent epigenetic modifications in the developing brain. To assess these changes, comprehensive transcriptome analyses were performed using the SurePrint microarray technique from Agilent Technology. Hippocampus tissues obtained from two marmosets reared by their parents and three marmosets that underwent human hand-rearing were utilized in the study. The selected marmosets, classified as young marmosets, were within the age range of 13 to 19 months. By comparing the gene expression profiles between the human hand-reared and parent-reared young marmosets, we identified 613 genes that exhibited significant differential expression in the hippocampus. Out of these genes, 425 were found to be significantly upregulated, and 188 genes were significantly downregulated in the human hand-reared marmosets (Fig. 2a). The criteria used for determining differential expression were fold change greater than 2.0 for upregulated genes and fold change less than -2.0 for downregulated genes, with a significance level of P < 0.05. These findings suggest that early parental deprivation can cause significant alterations in gene expression patterns in the hippocampus, potentially impacting various molecular processes and pathways involved in neuronal function and development.

To identify the biological processes associated with gene expression changes in early parental deprivation, we conducted functional annotation analysis using the DAVID (Database for Annotation, Visualization, and Integrated Discovery) tools^30,31^. Among the 425 genes that displayed upregulation in human hand-reared marmosets, two Gene Ontology (GO) clusters with high enrichment scores were identified (Table.1). The primary cluster, exhibiting the highest enrichment, was associated with the Neuroactive-Ligand Receptor Interaction Pathway. Additionally, Gene Set Enrichment Analysis (GSEA)^32,33^ indicated a correlation between the upregulated genes in human hand-reared marmosets and the Neuroactive-Ligand Receptor Interaction Pathway (Fig. 2b). Within this pathway, GABA Receptor family (*GABRR1*, *GABRG1*, *GABRA2*, *GABRA3*, *GABRB1*), and cholinergic receptor family (*CHRNA5, CHRNA3, CHRNA10*, *CHRM5*) and *OXTR* showed notable upregulation in human hand-reared marmosets (Fig. 2c). Conversely, no GO clusters with significantly high enrichment scores were identified among the 188 genes displaying downregulation in human hand-reared marmosets. This finding suggests that the genes upregulated in human hand-reared marmosets are involved in neuroactive ligand-receptor interactions, which may play a significant role in the short-term consequences of early parental deprivation.

**Table 1:** Gene ontology (GO) enrichment analysis toward the 425 genes that were upregulated in human hand-reared marmosets.

Cluster 1	Enrichment score 4.81	Count	P-value	FDR-adjusted P-value
KEGG PATHWAY	Neuroactive ligand-receptor interaction	28	3.2E−9	7.6E−7
UP_KW_MOLECULAR_FUNCTION	Receptor	45	5.7E−8	3.3E−6
UP_SEQ_FEATURE	DOMAIN:G-protein coupled receptors family 1 profile	26	1.9E−7	2.4E−5
INTERPRO	GPCR, rhodopsin-like, 7TM	24	6.7E−6	4.4E−3
INTERPRO	G protein-coupled receptor, rhodopsin-like	23	1.3E−5	4.4E−3
UP_KW_MOLECULAR_FUNCTION	Transducer	28	6.1E−5	1.8E−3
SMART	SM01381	9	3.6E−4	5.9E−2
UP_KW_MOLECULAR_FUNCTION	G protein-coupled receptor	16	1.4E−2	2.6E−1
UP_KW_CELLULAR_COMPONENT	Cell membrane	23	5.9E−2	4.9E−1

Cluster 2	Enrichment score 3.18	Count	P-value	FDR-adjusted P-value
UP_SEQ_FEATURE	TRANSMEM:Helical	127	3.8E−9	9.5E−7
GOTERM_CC_DIRECT	Integral component of membrane	111	4.4E−4	4.7E−2
UP_KW_CELLULAR_COMPONENT	Membrane	137	1.3E−2	1.6E−1
UP_KW_DOMAIN	Transmembrane	94	4.2E−2	3.1E−1
UP_KW_DOMAIN	Transmembrane helix	66	1.4E−1	7.1E−1

### Identification of potential pathways and processes involved in the long-term consequences of early parental deprivation

**Figure 3:**
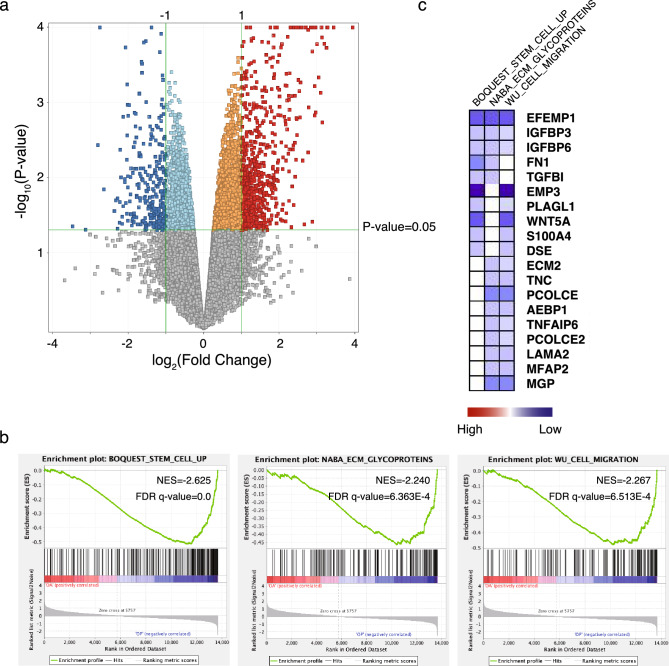
Identification of potential pathways and processes involved in the long-term consequences of early parental deprivation. **(a)** Volcano plot of human hand-reared aged marmosets vs parent-reared aged marmosets. In the plot, 381 genes that exhibited upregulation in the human hand-reared marmosets were shown in red color (fold change > 2.0, P < 0.05). On the other hand, 186 genes that displayed downregulation were shown in blue color (fold change < −2.0, P < 0.05). **(b)** GSEA of the BOQUEST STEM CELL UP, NABA ECM GLYCOPROTEINS, and WU CELL MIGRATION enriched in human hand-reared marmosets compared to parent-reared aged marmosets. **(c)** Heat Map illustrates the partially or completely overlapped genes among BOQUEST STEM CELL UP, NABA ECM GLYCOPROTEINS, and WU CELL MIGRATION gene sets. The intensity of the blue color indicates the degree of decreased expression.

To investigate the persistence of gene expression changes observed in human hand-reared young marmosets into adulthood, we conducted comprehensive gene expression analyses in marmosets ranging from 88 to 164 months of age. By comparing the gene expression profiles between these two age groups, we aimed to assess whether the expression changes associated with early parental deprivation were maintained or altered in adulthood. This analysis provides insights into the long-term consequences of early parental deprivation on gene expression patterns in the hippocampus and can help us understand the molecular mechanisms underlying the lasting effects of early-life experiences. By comparing the gene expression profiles between human hand-reared and parent-reared aged marmosets (between 88 to 164 months of age), we identified 567 genes that showed significant differential expression in the hippocampus. Among these genes, 381 genes were found to be significantly upregulated (fold change > 2.0, P < 0.05) in the human hand-reared marmosets, while 186 genes were significantly downregulated (fold change < − 2.0, P < 0.05) in the human hand-reared marmosets (Fig. 3a). Furthermore, we performed GSEA to gain insights into the biological processes associated with these gene expression changes in human hand-reared aged marmosets. The analysis revealed that the downregulated genes in human hand-reared marmosets were associated with three gene sets: BOQUEST STEM CELL UP, NABA ECM GLYCOPROTEINS, and WU CELL MIGRATION (Fig. 3b). Figure 3c illustrates the partially or completely overlapped genes among these three gene sets. Notably, within these gene sets, several genes are of particular interest. The *EFEMP1* gene, associated with dementia^34^, exhibited downregulation in human hand-reared marmosets, suggesting a potential link between its expression and the observed cognitive effects^19^. Furthermore, the *IGFBP3/6* and *WNT5a* genes, known for their involvement in neuronal differentiation and proliferation^35,36^, showed downregulation in human hand-reared marmosets. Additionally, the gene *S100A4*, which has a neuroprotective pro-survival effect on neurons during brain injury^37^, was identified among the downregulated genes. Its downregulation suggests a potential reduction in the neuroprotective response, potentially contributing to increased vulnerability to adverse effects following early parental deprivation. Conversely, GSEA did not reveal any significant correlations between the upregulated genes in human hand-reared marmosets and the potential pathway related to behavior or neural development. These findings provide valuable insights into the molecular mechanisms underlying the consequences of early parental deprivation in human hand-reared aged marmosets. The identified gene sets and associated genes shed light on the potential pathways and processes involved in the observed gene expression changes and associated cognitive and behavioral alterations.

### Identification of genes continuously affected by early parental deprivation throughout life

In this study, we delved into the gene expression profiles within the hippocampus of both young and aged human hand-reared marmosets, comparing them to their parent-reared counterparts. Our objective was to understand the persistent impact of early parental deprivation on gene expression throughout their lives.

Upon analyzing the gene expression data, we identified 613 genes that exhibited significant differential expression in the hippocampus of human hand-reared young marmosets and 567 genes in human hand-reared aged marmosets, when compared to parent-reared age-matched marmosets. To explore the overlap between these two developmental stages, we performed a Venn diagram analysis.

**Figure 4:**
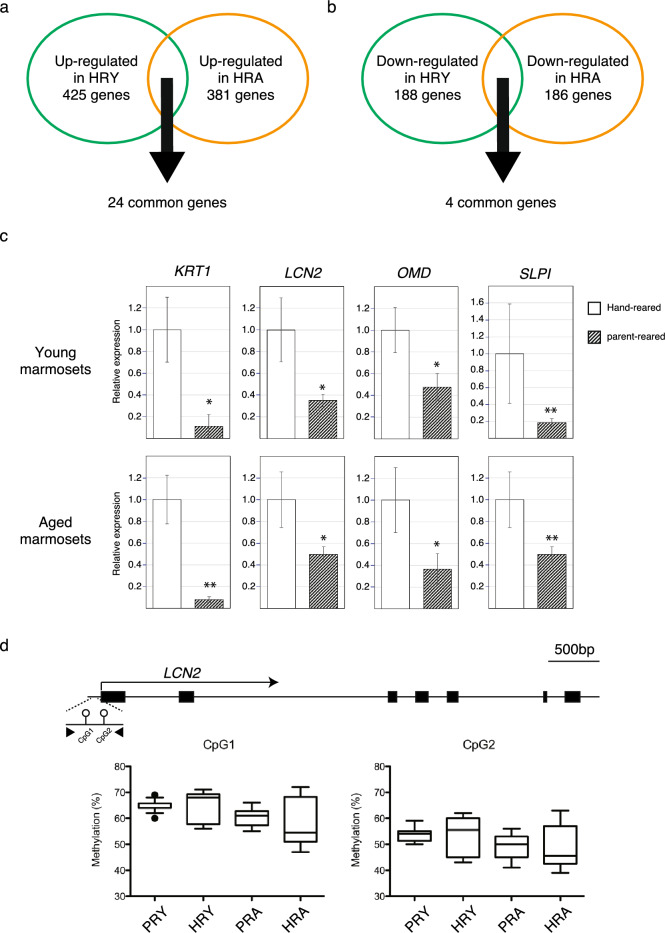
Identification of genes continuously affected by early parental deprivation throughout life. **(a)** Venn diagram for detecting commonly upregulated genes in both human hand-reared young marmosets and aged marmosets.** (b)** Venn diagram for detecting commonly downregulated genes in both human hand-reared young marmosets and aged marmosets.** (c)** Real-time PCR analysis to validate the microarray results for expression levels of *KRT1*, *LCN2*, *OMD*, and *SLPI* genes in human hand-reared marmosets compare to parent-reared marmosets. These genes were found to be down-regulated in both young and aged stages. *p < 0.05, **P < 0.01** (d)** DNA methylation analysis of the promoter region of the *LCN2* gene. Genomic map of the *Lcn2* gene locus; scale bar = 500 bp. The positions of primers used for pyrosequencing are indicated by arrowheads. *HRY* *h*and-*r*eared *y*oung marmoset, *PRY* *p*arent-*r*eared *y*oung marmoset, *HRA* *h*and-*r*eared *a*ged marmoset, *PRA* *p*arent-*r*eared *a*ged marmoset.

The Venn diagram analysis revealed that among the differentially expressed genes, only 24 genes were up-regulated (Fig. 4a, Table 2) and 4 genes were down-regulated in both young and aged stages (Fig. 4b, Table 3). This suggests that there is a limited overlap in the gene expression changes associated with early parental deprivation across the two developmental stages.

**Table 2:** Commonly up-regulated genes list in both human hand-reared young marmosets and aged marmosets.

Gene symbol	Description	Gene type	FC(YAvsYP)	FC(OAvsOP)
5S_rRNA	5S ribosomal RNA	rRNA	2.50	2.46
C6	Complement C6	Protein coding	2.01	3.57
FAM227A	Family with sequence similarity 227 member A	Protein coding	2.02	2.63
HORMAD1	HORMA domain containing 1	Protein coding	5.09	3.43
IDO2	Indoleamine 2,3-dioxygenase 2	Protein coding	4.15	2.41
MBL2	Mannose binding lectin 2	Protein coding	2.31	3.09
MCMDC2	Minichromosome maintenance domain containing 2	Protein coding	2.77	2.04
PTGIR	Prostaglandin l2 (prostacyclin) receptor	Protein coding	2.37	2.05
RPL31	Ribosomal protein L31	Protein coding	2.45	2.47
SCGB3A2	Secretoglobin family 3A member 2	Protein coding	2.49	2.48
SLC18A2	Solute carrier family 18 member A2	Protein coding	2.69	3.62
SLC23A3	Solute carrier family 23 member 3	Protein coding	2.28	2.82
SNORA11	Small nucleolar RNA SNORA11	snoRNA	4.54	2.21
SNORD112	Small nucleolar RNA SNORA112	snoRNA	2.20	2.10
SNORD113	Small nucleolar RNA SNORA113	snoRNA	2.18	2.23
snoU109	Small nucleolar RNA U109	snoRNA	4.05	2.09
snoU13	Small nucleolar RNA U13	snoRNA	2.24	2.79
TAS2R5	Taste 2 receptor associated factor 5	Protein coding	2.55	2.33
TBXAS1	Thromboxane A synthase 1	Protein coding	4.25	2.46
TRAF5	TNF receptor associated factor 5	Protein coding	3.18	2.03
U1	U1 spliceosomal RNA	snRNA	2.51	2.22
U6	U6 spliceosomal RNA	U6	2.59	3.01
VTN	Vitronectin	Protein coding	3.32	4.01
Y_RNA	Y RNA	Misc RNA	2.70	3.11

**Table 3:** Commonly down-regulated genes list in both human hand-reared young marmosets and aged marmosets.

Gene symbol	Description	Gene type	FC(YAvsYP)	FC(OAvsOP)
KRT1	Keratin 1	Protein coding	−12.00	−2.14
LCN2	Lipocalin 2	Protein coding	−5.70	−2.63
OMD	Osteomodulin	Protein coding	−2.77	−2.48
SLPI	Secretory leukocyte peptidase inhibitor	Protein coding	−2.01	−2.68

To further validate our Microarray analysis findings, we conducted real-time PCR analysis to assess the expression levels of *KRT1*, *LCN2*, *OMD*, and *SLPI* genes in the hippocampus, which were found to be down-regulated in both young and aged stages (Fig. 4b, Table 3). The results of the real-time PCR analysis confirmed that the expression levels of these genes were consistently affected by early parental deprivation, which was consistent with the findings obtained from the microarray analysis (Fig. 4c). In addition to the gene expression analysis, we also conducted a DNA methylation analysis of the promoter region of the *LCN2* gene. This analysis was performed to investigate the potential involvement of DNA methylation, specifically in the promoter region of *LCN2*, which is known to be one of the target genes of MECP2^38^. MECP2 indeed functions as a transcription factor capable of binding to methylated CpG sites, thereby influencing the regulation of downstream gene expression, either positively or negatively. By examining the DNA methylation patterns in the promoter region of *LCN2* using bisulfite sequencing, we aimed to explore the potential epigenetic mechanisms underlying the continuous alterations in gene expression observed in response to early parental deprivation. However, the results of the DNA methylation analysis did not provide insights into the regulatory mechanisms involved in the persistent changes in *LCN2* gene expression and their association with early life experiences and parental deprivation (Fig. 4d). We did not observe any significant differences in DNA methylation levels in the hippocampus of human hand-reared young and aged marmosets, in comparison to parent-reared age-matched marmosets. It is important to note that while DNA methylation is a well-known epigenetic mechanism, it is not the sole determinant of gene expression regulation. Other epigenetic modifications, such as histone modifications or non-coding RNA molecules, could be involved in mediating the effects of early parental deprivation on gene expression. Further investigations into alternative epigenetic mechanisms and regulatory processes may provide a more comprehensive understanding of the underlying mechanisms involved in the observed alterations in gene expression.

## Discussion

This study represents the first investigation into the effects of early parental deprivation on gene expression changes in in common marmosets at two developmental stages, Young and Aged, using microarray analysis. While previous research has predominantly concentrated on exploring the effects of maternal separation in rodent models^9,10,24,25^, it is crucial to note that the differences in the central stress response system between rodents and primates do not fully capture the complex dynamics of parent loss, abuse, or neglect observed in human populations^12,13^. Furthermore, studies conducted with rodents or non-human primates have demonstrated that repeated maternal separation, typically occurring for several hours per day during the initial or second week of infancy, can trigger acute stress responses^9,10,14–17,24,25^. Conversely, non-human primates separated from their parents shortly after birth tend to exhibit a less pronounced acute stress response^21^. Interestingly, this study observed no significant differences in body weight between human hand-reared and parent-reared marmosets from birth (Fig. 1c). However, marmosets experiencing repeated parental deprivation in infancy displayed reduced body weight and elevated levels of ACTH and cortisol compared to the control group as previously described ^14–16^. These findings highlight variations in the maternal separation paradigm and emphasize the importance of considering these differences when investigating the impact of early life experiences on stress physiology and behavior. In the present study, we observed upregulation of genes belonging to the Neuroactive-Ligand Receptor Interaction Pathway, including the *OXTR* gene, in young marmosets that underwent human hand-rearing. Previous experiments conducted on rhesus macaques have reported a decrease in the expression of the *OXTR* gene and changes in epigenetic modifications^21^, which contradicts the findings of the present study. However, it is important to note that the discrepancy in results may be attributed to the different developmental stages that were analyzed. In our marmoset experiments, similar to the rhesus macaques, we observed a slight reduction in the expression of the *OXTR* gene during the Aged stage. These findings highlight the dynamic nature of gene expression in response to early life experiences and indicate that the effects of early parental deprivation on gene expression may vary across different stages of development. Additionally, we identified a set of genes that showed consistent alterations across developmental stages and were continuously affected by early parental deprivation. It is particularly interesting that some of these genes are involved in the development of neurodevelopmental disorders and neuronal function.

The up-regulated gene *SLC18A2*(*VMAT2*) functions by packaging dopamine into vesicles in synaptic terminal neurons and releasing it during neurotransmission^39^. Rats raised in social isolation have been shown to exhibit increased ethanol and cocaine intake, along with enhanced dopamine and VMAT2 levels, which may contribute to a heightened vulnerability to addiction^40^. This suggests that abnormal dopamine release in human hand-reared marmosets could be associated with an increased risk of addiction.

On the other hand, *TAS2R5*, another up-regulated gene, has been found to exhibit higher gene expression during manic states based on analysis of peripheral blood samples in bipolar disorder^41^. This gene is involved in taste perception and may play a role in the manifestation of manic symptoms.

In contrast, the three down-regulated genes targeted in this study were *LCN2*, *SLPI*, and *OMD*, all of which encode extracellular secretory proteins. Secretory leukocyte protease inhibitor (SLPI) is a serine protease inhibitor with anti-inflammatory and antibacterial properties, and it promotes wound healing^42^. *Slpi* null mutant mice show reduced regeneration of posterior column axons in response to sciatic nerve injury^43^, suggesting that its decreased expression in this study may contribute to abnormal behavior due to impaired stress response and neuronal inflammation rescue. Osteomodulin (OMD) is a gene localized to extracellular exosomes and is believed to regulate cell adhesion and bone calcification^44^. A study related to psychiatric disorders found decreased expression of the *OMD* gene in amygdala gene expression analysis of children born after exposure to valproic acid (VPA) during the 12th day of gestation^45^. The precise mechanism by which this occurs is not yet known, but it suggests that OMD may have an impact on brain function. LCN2 is a secreted protein involved in innate immunity and has been shown to regulate spine morphology and suppress neuronal excitability^46^. *Lcn2* knockout (KO) mice exhibit anxiety and depression-like behavior, cognitive dysfunction, and changes in hippocampal brain cell structure^47^. Decreased expression of *Lcn2* has also been observed in a mouse model of Rett syndrome^38^. Although the exact mechanism of reduced *LCN2* expression is still unclear, these findings suggest that LCN2 may be involved in developmental disorders, such as Rett syndrome, and potentially plays a role in developmental abnormalities observed in human hand-reared marmosets. Overall, the analysis of differentially expressed genes in this study provides insights into the potential molecular mechanisms underlying the behavioral abnormalities observed in young marmosets subjected to human hand-rearing as described previously^19,20^. However, it is important to note that this study exclusively used male samples, potentially limiting the generalizability of the identified gene expression changes. To address this gap, future studies should explore whether similar alterations in gene expression occur in female samples, accounting for factors such as the menstrual cycle and other relevant variables. Furthermore, among the human hand-reared marmosets analyzed, some were offspring of human hand-reared mother marmosets, which raises concerns regarding potential effects (Fig. 1b). Despite this, any epigenetic mutations occurring in the parental generation are unlikely to be passed on to the offspring. Nevertheless, owing to the unverifiable breeding history of the grandparents in this study, definitive confirmation is not possible. If the grandparents were also human hand-reared, there remains a possibility that epigenetic mutations might have been transmitted to the individuals under examination in this study. The study highlights the long-term effects of the nurturing environment during infancy on gene expression. While stress is commonly associated with early life experiences, this particular study did not directly address stress as a variable. However, it is evident that the nurturing environment during infancy influenced gene expression in the hippocampus. Further investigations are needed to explore various aspects of the human hand-rearing paradigm, including the specific cellular targets affected by the nurturing environment.

In order to gain a comprehensive understanding of the human hand-reared marmoset brain, it will be essential to incorporate MRI imaging analysis^48^. This imaging technique can provide insights into the structural and functional characteristics of the brain, offering valuable information about how the nurturing environment impacts brain development.

Overall, further studies are warranted to elucidate the effects of the nurturing environment during infancy. By conducting comprehensive investigations, we can better understand the underlying mechanisms and implications of early life experiences on gene expression, stress physiology, and brain structure and function in human hand-reared marmosets. This knowledge may pave the way for exploring gene therapy as a potential intervention for individuals dealing with developmental disorders, an area of growing significance in research and healthcare.

## Methods

### Animals

All animal experiments were approved by the Institutional Animal Experimentation Committee of the Central Institute for Experimental Animals (CIEA: 20049A) and conducted in compliance with the CIEA Standard Guidelines. These guidelines align with the recommendations for the proper conduct of animal experiments established by the Science Council of Japan and were reported in accordance with the ARRIVE guidelines.

The marmosets used in this study were procured from CLEA Japan, a facility accredited by the Japanese Society for Laboratory Animals Resources (certification number: 22-041). All animals received appropriate care under the supervision of the Institutional Animal Care and Use Committee of CLEA Japan, Inc. until their transfer to the CIEA (Central Institute for Experimental Animals). Crucially, the dimensions of the housing enclosures (W550 mm, D390 mm, H700 mm) were meticulously designed to align with the guidelines set forth by the US National Research Council. This adherence underscores our commitment to providing an environment that caters to the animals' well-being. The animal room maintained a temperature range of 23–29 °C, humidity between 30 and 70%, and a 12-h light–dark cycle (lights on from 8:00 to 20:00). The marmosets had access to pellet-type CMS-1M feed (CLEA Japan Inc) and water ad libitum. Enrichment strategies were thoughtfully implemented, including the provision of wooden sticks and boards within the cages, affording the marmosets the opportunity to freely engage in climbing and resting behaviors. Notably, the enclosures were designed to facilitate visual contact and audible communication between marmosets housed in different cages.

This study included eleven male marmosets, comprising six human hand-reared marmosets and five parent-reared marmosets. Hand-rearing was performed for neonates that were excluded from parental care when three or four offspring were born.

Brain tissue sampling was performed when the marmosets were 13–19 months old as young marmosets and 88–164 months old as aged marmosets.

### Hand-rearing

Hand-rearing was conducted at CLEA Japan Inc. When three or more neonates were born from the same litter, two remained with the mother, and the remaining individuals underwent human hand-rearing. Traditionally, we assigned larger, stronger offspring to parental care, while smaller or weaker ones were hand-reared at our facility. However, our recent findings demonstrate that parent marmosets effectively care for their young, regardless of size or vitality. Consequently, to enhance the survival rate of marmosets under human-hand rearing, we deliberately choose heavier and sturdier individuals for this care. Our hand-rearing protocol involved specific steps. Shortly after birth, the neonates were separated from their parents and individually placed in small plastic cages measuring 150 mm × 215 mm × 120 mm. These cages were located in a separate animal room, ensuring no visual or auditory contact with their parents, within a standard incubator. The incubator maintained a temperature range of 27–33 °C, humidity between 40 and 80%, and a 12-h light–dark cycle (lights on from 7:00 to 19:00). Each plastic cage was equipped with various surrogates such as soft diaper sheets that the neonate could wrap and cling to, a heating pad covered with artificial fur, to ensure the neonates' sense of security.

Despite individual housing from postnatal day 0 to 30, the setup facilitated visual and vocal contact among neonates in neighboring cages. At around 30 days of age, the neonates were transferred from the incubator to slightly larger cages measuring 210 mm × 270 mm × 300 mm, where three marmosets lived together. By 50 days of age, five animals resided in a mid-sized cage measuring 550 mm × 780 mm × 700 mm.

As the marmosets matured, the feeding frequency gradually decreased, allowing them to consume larger quantities of milk per feeding. The feeding schedule adapted as the marmosets matured. It started with four daily feedings (0.9 ml to 1.4 ml) from days 0 to 7, with an increase in formula concentration. From days 7 to 27, three feedings (1.6 ml to 5.6 ml) were supplemented with banana lining and vitamin D3, alongside a gradual increase in formula concentration. From days 28 to 59, milk volume increased (8.0 ml to 10 ml), and additional food was introduced twice daily. Post-60 days, each feeding comprised 12 ml of formula, accompanied by once-daily solid feed. Throughout these periods, human caregivers interacted with the marmosets solely during suckling times.

Care was provided by human caregivers until the marmosets reached three months of age, at which point they were successfully weaned. Following this milestone, two same-aged males were housed together in cages measuring 550 mm × 390 mm × 700 mm.

Our care approach strictly adhered to the guidelines outlined in the 'Common Marmoset Management Work Manual,' developed under the supervision of the Institutional Animal Care and Use Committee of CLEA Japan Inc.

### Brain sampling

Brain tissue sampling was performed as follows. The marmosets were first anesthetized with a mixture of 0.04 mg/kg medetomidine, 0.4 mg/kg midazolam, 0.4 mg/kg butorphanol, and 0.4 mg/kg butanol. Deep anesthesia was induced by isoflurane inhalation (0.5–3%). To confirm complete loss of pain response, skin was gently pinched at several sites using tweezers. After confirming the absence of pain response, blood was drawn from the inferior vena cava, and the marmoset was euthanized. The skull was quickly removed, the hippocampus was dissected, and the brain was flash-frozen in liquid nitrogen.

### Microarray analysis

Total RNA was extracted from eleven marmoset hippocampus tissues using the TRIzol Reagent with the PureLink RNA Mini Kit (Thermo Fisher Scientific, USA). RNA quality was assessed using the Agilent 2200 TapeStation (Agilent, USA). RNA samples with a RIN value greater than 5.7 and 28S/18S ratio greater than 0.9 were selected for subsequent experiments (Supplementary Table S2). The microarray analysis was performed following the manufacturer's protocol. Briefly, 0.2 µg of total RNA was used to prepare Cyanine-3 (Cy3)-labeled cRNA using the Low Input Quick Amp Labeling Kit (Agilent, USA), followed by RNAeasy column purification (Qiagen, USA). Dye incorporation and cRNA yield were measured using the NanoDrop ND-1000 Spectrophotometer. The fragmented Cy3-labeled cRNA was then hybridized to the Marmoset microarray 8x60K (G4858A#84626, Agilent, USA). After hybridization, the microarrays were washed and scanned using the Agilent DNA Microarray Scanner. The scanned images were analyzed with Feature Extraction Software 11.0.1.1 (Agilent, USA) using default parameters, and the obtained data were normalized and filtered using three filters with GeneSpring software 14.9 (Agilent, USA).

The microarray data have been deposited in the Gene Expression Omnibus (GEO) database under the accession number GSE236481.

### Real-time RT-qPCR

The total RNA was reverse transcribed into cDNA using the PrimeScript IV RT Master Mix (Takara, Japan) following the manufacturer's instructions. Real-Time PCR analysis was performed using the SsoAdvanced Universal SYBR Green Supermix (Bio-Rad, USA) and the CFX Connect Real-Time PCR System (Bio-Rad, USA). The thermal cycling conditions consisted of 1 cycle at 98 °C for 30 s, followed by 40 cycles at 98 °C for 10 s and 60 °C for 30 s. The quality and specificity of the PCR reactions were confirmed by melting curve analysis. The primer sequences are listed in Supplementary Table S1. The hypoxanthine guanine phosphoribosyl transferase (Hprt) gene was used as the internal control. The relative gene expression of target genes was calculated using the ΔΔCt method. Statistical analysis was performed using Student's t-test, and p values < 0.05 were considered statistically significant.

### DNA methylation analysis

DNA was isolated from the hippocampus tissue using the classic phenol–chloroform method. Briefly, the tissue sample was incubated in lysis buffer (100 mM Tris–HCl pH 7.5, 20 mM EDTA, 165 mM NaCl, 1.0% SDS, 70 μg/ml proteinase K) at 55 °C overnight. The resulting lysate was treated with an equal volume of a phenol/chloroform/isoamyl alcohol mixture (Nacalai, Japan) and purified through ethanol precipitation. The concentration of the extracted DNA was measured using the NanoDrop ND-1000 Spectrophotometer.

Subsequently, 1 μg of DNA was subjected to bisulfite conversion using the EZ DNA Methylation-Gold Kit (Zymo Research, USA) following the manufacturer's protocols. The bisulfite-treated DNA was then used for PCR amplification using the PyroMark PCR kit (Qiagen, USA) according to the manufacturer's protocols. The pyrosequencing reaction was performed on a PyroMark Q24 instrument (Qiagen, USA). The resulting data were analyzed and quantified using the PyroMark Q24 software version 2.0.8 (Qiagen, USA). The PCR and pyrosequencing primers used were listed in Table S1, designed with the PyroMark Assay Design software v2.0.2.5 (Qiagen, USA).

### Gene ontology and gene set enrichment analysis

DAVID was utilized to identify and enrich the biological attributes, including biological processes (BP), cellular components (CC), and molecular functions (MF), of the differentially expressed genes (DEGs). The Kyoto Encyclopedia of Genes and Genomes (KEGG) pathway (http://www.genome.jp/) was used to identify significant pathways. A significance cutoff of P < 0.05 was applied for enrichment analysis. Functional annotations were predicted using the gene set enrichment analysis (GSEA) software v4.3.2 downloaded from the Broad Institute (https://www.gsea-msigdb.org/gsea/index.jsp). The thresholds for significance were determined through permutation analysis (1000 permutations). Significant enrichments were defined as nominal p values < 0.05 and FDR q < 25%, as suggested by GSEA. Enrichment plots were generated from the GSEA reports.

### Statistical analysis

All data are presented using dot plots, box plots, and expressed as mean ± standard error of the mean (SEM) in Real-Time RT-qPCR and DNA methylation analysis. The Student's t-test was employed for comparisons, and a p-value less than 0.05 was deemed statistically significant.

### Supplementary Information

Supplementary Tables.

## References

[1] Kendler KS, Neale MC, Kessler RC, Heath AC, Eaves LJ (1996). Childhood parental loss and alcoholism in women: A causal analysis using a twin-family design. Psychol. Med..

[2] Heim C, Nemeroff CB (2001). The role of childhood trauma in the neurobiology of mood and anxiety disorders: Preclinical and clinical studies. Biol. Psychiatry.

[3] Holmes A (2005). Early life genetic, epigenetic and environmental factors shaping emotionality in rodents. Neurosci. Biobehav. Rev..

[4] McEwen BS (2008). Understanding the potency of stressful early life experiences on brain and body function. Metabolism.

[5] Cameron NM (2005). The programming of individual differences in defensive responses and reproductive strategies in the rat through variations in maternal care. Neurosci. Biobehav. Rev..

[6] Harlow HF, Dodsworth RO, Harlow MK (1965). Total social isolation in monkeys. Proc. Natl. Acad. Sci. USA.

[7] Phillips R (2013). The sacred hour: Uninterrupted skin-to-skin contact immediately after birth. Newborn Infant Nurs. Rev..

[8] Danese A, McEwen BS (2012). Adverse childhood experiences, allostasis, allostatic load, and age-related disease. Physiol. Behav..

[9] Weaver IC (2004). Epigenetic programming by maternal behavior. Nat. Neurosci..

[10] Zhu Y (2017). Enhanced neuroinflammation mediated by DNA methylation of the glucocorticoid receptor triggers cognitive dysfunction after sevoflurane anesthesia in adult rats subjected to maternal separation during the neonatal period. J. Neuroinflamm..

[11] Gray JD, Kogan JF, Marrocco J, McEwen BS (2017). Genomic and epigenomic mechanisms of glucocorticoids in the brain. Nat. Rev. Endocrinol..

[12] Sánchez MM, Young LJ, Plotsky PM, Insel TR (1999). Autoradiographic and in situ hybridization localization of corticotropin-releasing factor 1 and 2 receptors in nonhuman primate brain. J. Comp. Neurol..

[13] Kaiser T, Feng G (2015). Modeling psychiatric disorders for developing effective treatments. Nat. Med..

[14] Pryce CR, Palme R, Feldon J (2002). Development of pituitary-adrenal endocrine function in the marmoset monkey: infant hypercortisolism is the norm. J. Clin. Endocrinol. Metab..

[15] Dettling AC, Feldon J, Pryce CR (2002). Repeated parental deprivation in the infant common marmoset (*Callithrix jacchus*, primates) and analysis of its effects on early development. Biol. Psychiatry.

[16] Pryce CR, Dettling AC, Spengler M, Schnell CR, Feldon J (2004). Deprivation of parenting disrupts development of homeostatic and reward systems in marmoset monkey offspring. Biol. Psychiatry.

[17] Arabadzisz D (2010). Primate early life stress leads to long-term mild hippocampal decreases in corticosteroid receptor expression. Biol. Psychiatry.

[18] Suomi SJ, Leroy HA (1982). In memoriam: Harry F. Harlow (1905–1981). Am. J. Primatol..

[19] Ziegler TE, Stein FJ, Sis RF, Coleman MS, Green JH (1981). Supplemental feeding of marmoset (*Callithrix jacchus*) triplets. Lab Anim. Sci..

[20] Gultekin YB, Hage SR (2018). Limiting parental interaction during vocal development affects acoustic call structure in marmoset monkeys. Sci. Adv..

[21] Baker M (2017). Early rearing history influences oxytocin receptor epigenetic regulation in rhesus macaques. Proc. Natl. Acad. Sci. USA.

[22] Law AJ (2009). Early parental deprivation in the marmoset monkey produces long-term changes in hippocampal expression of genes involved in synaptic plasticity and implicated in mood disorder. Neuropsychopharmacology.

[23] Caldji C, Francis D, Sharma S, Plotsky PM, Meaney MJ (2000). The effects of early rearing environment on the development of GABAA and central benzodiazepine receptor levels and novelty-induced fearfulness in the rat. Neuropsychopharmacology.

[24] Pryce CR, Dettling A, Spengler M, Spaete C, Feldon J (2004). Evidence for altered monoamine activity and emotional and cognitive disturbance in marmoset monkeys exposed to early life stress. Ann. N.Y. Acad. Sci..

[25] Kikusui T, Kiyokawa Y, Mori Y (2007). Deprivation of mother-pup interaction by early weaning alters myelin formation in male, but not female, ICR mice. Brain Res..

[26] van Leeuwen EJ, Mulenga IC, Chidester DL (2014). Early social deprivation negatively affects social skill acquisition in chimpanzees (*Pan troglodytes*). Anim. Cogn..

[27] Bouchet H (2022). Early life experience and sex influence acoustic repertoire use in wild-born, but hand-reared, captive cheetahs (*Acinonyx jubatus*). Dev. Psychobiol..

[28] Sasaki E (2009). Generation of transgenic non-human primates with germline transmission. Nature.

[29] Pryce CR (1993). The regulation of maternal behaviour in marmosets and tamarins. Behav. Process..

[30] da Huang W, Sherman BT, Lempicki RA (2009). Systematic and integrative analysis of large gene lists using DAVID bioinformatics resources. Nat. Protoc..

[31] Sherman BT (2022). DAVID: A web server for functional enrichment analysis and functional annotation of gene lists (2021 update). Nucleic Acids Res..

[32] Subramanian A (2005). Gene set enrichment analysis: A knowledge-based approach for interpreting genome-wide expression profiles. Proc. Natl. Acad. Sci. USA.

[33] Mootha VK (2003). PGC-1alpha-responsive genes involved in oxidative phosphorylation are coordinately downregulated in human diabetes. Nat. Genet..

[34] McGrath ER (2022). Plasma EFEMP1 is associated with brain aging and dementia: The Framingham Heart Study. J. Alzheimers Dis..

[35] Bunn RC, King WD, Winkler MK, Fowlkes JL (2005). Early developmental changes in IGF-I, IGF-II, IGF binding protein-1, and IGF binding protein-3 concentration in the cerebrospinal fluid of children. Pediatr. Res..

[36] Arredondo SB (2020). Wnt5a promotes differentiation and development of adult-born neurons in the hippocampus by noncanonical Wnt signaling. Stem Cells.

[37] Dmytriyeva O (2012). The metastasis-promoting S100A4 protein confers neuroprotection in brain injury. Nat. Commun..

[38] Delépine C (2015). Astrocyte transcriptome from the Mecp 2(308)-truncated mouse model of Rett syndrome. Neuromol. Med..

[39] Caudle WM (2007). Reduced vesicular storage of dopamine causes progressive nigrostriatal neurodegeneration. J. Neurosci..

[40] Karkhanis AN (2019). Chronic social isolation stress during peri-adolescence alters presynaptic dopamine terminal dynamics via augmentation in accumbal dopamine availability. ACS Chem. Neurosci..

[41] Lee YC (2019). Transcriptome changes in relation to manic episode. Front. Psychiatry.

[42] Xie M (2022). Identifying crucial biomarkers in peripheral blood of schizophrenia and screening therapeutic agents by comprehensive bioinformatics analysis. J. Psychiatr. Res..

[43] Hannila SS (2013). Secretory leukocyte protease inhibitor reverses inhibition by CNS myelin, promotes regeneration in the optic nerve, and suppresses expression of the transforming growth factor-beta signaling protein Smad2. J. Neurosci..

[44] Skenteris NT (2022). Osteomodulin attenuates smooth muscle cell osteogenic transition in vascular calcification. Clin. Transl. Med..

[45] Oguchi-Katayama A, Monma A, Sekino Y, Moriguchi T, Sato K (2013). Comparative gene expression analysis of the amygdala in autistic rat models produced by pre- and post-natal exposures to valproic acid. J. Toxicol. Sci..

[46] Mucha M (2011). Lipocalin-2 controls neuronal excitability and anxiety by regulating dendritic spine formation and maturation. Proc. Natl. Acad. Sci. USA.

[47] Ferreira AC (2013). Lipocalin-2 is involved in emotional behaviors and cognitive function. Front. Cell Neurosci..

[48] Seki F (2017). Developmental trajectories of macroanatomical structures in common marmoset brain. Neuroscience.

