# Supplementary material for: Early parental deprivation during primate infancy has a lifelong impact on gene expression in the male marmoset brain
Source: Sci Rep. 2024 Jan 3;14:330. doi: 10.1038/s41598-023-51025-z (PMC10764730; doi:10.1038/s41598-023-51025-z)
Supplement: Supplementary file 1 — Supplementary Tables. [file 41598_2023_51025_MOESM1_ESM.docx]

**Supplementary Table S1. Primer information**

| **Primer name 5’-3’ Sequence Description** | | |
| --- | --- | --- |
| KRT1_forward | CAGACATGGGGATAGCGTGA | KRT1 qPCR |
| KRT1_reverse | CACTGATGGACTGCTGCAAG |  |
| LCN2_forward | CGCTGAAGATGTATGCCACC | LCN2 qPCR |
| LCN2_reverse | GTTGCCCAGCTTGAACTCTC |  |
| OMD_forward | TCAACCCCTGAAAACTAAAGCA | OMD qPCR |
| OMD_reverse | GCAATGCACTTTGACTCCAA |  |
| SLPI_forward | TGTTGTCCTGGCATTTGTCC | SLPI qPCR |
| SLPI_reverse | TCCGACTCACAGTGATTGGG |  |
| HPRT_forward | GCTGAGGATTTGGAAAGGGTG | HPRT qPCR |
| HPRT_reverse | GCTACAATGTGATGGCCTCC |  |
| LCN2_Me_forward | AAGGGTATAAAGAGTGTATAGAGG | LCN2 Methylation analysis |
| LCN2_Me_reverse(Biotin) | ACTTAACAAAATTTCTACCCCAAATCA |  |
| LCN2_Me_seq | TGTTTTTGTTAGAGGTGT |  |

**Supplementary Table S2. RNA quality information**

| **Sample_ID RIN (RNA Integrity Number) 28S/18S** | | |
| --- | --- | --- |
| I7500M | 7.5 | 1.6 |
| I7499M | 5.7 | 1.2 |
| I7535M | 7.8 | 1.6 |
| I7540M | 7.9 | 1.8 |
| I7514M | 7.2 | 1.6 |
| I6043M | 7.6 | 1.3 |
| I5728M | 8.3 | 1.7 |
| I3913M | 8.1 | 1.6 |
| I5352M | 6.5 | 1.3 |
| I4736M | 6.0 | 0.9 |
| X069M | 7.5 | 1.5 |
